# High Efficiency *In Vivo* Genome Engineering with a Simplified 15-RVD GoldyTALEN Design

**DOI:** 10.1371/journal.pone.0065259

**Published:** 2013-05-29

**Authors:** Alvin C. Ma, Han B. Lee, Karl J. Clark, Stephen C. Ekker

**Affiliations:** Department of Biochemistry and Molecular Biology, Mayo Clinic, Rochester, Minnesota, United States of America; University of Birmingham, United Kingdom

## Abstract

Transcription activator-like effector nucleases (TALENs) enable genome engineering in cell culture and many organisms. Recently, the GoldyTALEN scaffold was shown to readily introduce mutations in zebrafish (*Danio rerio*) and livestock through non-homologous end joining (NHEJ) and homology-directed repair (HDR). To deploy the GoldyTALEN system for high-throughput mutagenesis in model organisms, a simple design with high efficacy is desirable. We tested the *in vivo* efficacy of a simplified 15-RVD GoldyTALEN design (spacer between 13–20 bp and T nucleotide preceding each TALEN binding site) in zebrafish. All 14 tested TALEN pairs (100%) introduced small insertions and deletions at somatic efficacy ranging from 24 to 86%, and mutations were inheritable at high frequencies (18–100%). By co-injecting two GoldyTALEN pairs, inheritable deletions of a large genomic fragment up to 18 kb were successfully introduced at two different loci. In conclusion, these high efficiency 15-RVD GoldyTALENs are useful for high-throughput mutagenesis in diverse application including hypothesis testing from basic science to precision medicine.

## Introduction

Custom restriction enzymes including zinc finger nucleases (ZFNs) and transcription activator-like effector nucleases (TALENs) are valuable tools in genome editing (for review, see Carlson, D.F. et al [1]). Both encode a FokI nuclease catalytic domain fused with a customizable DNA binding domain that determines their targeting specificity. The ability of ZFNs to introduce targeted double-stranded breaks in a genome can be used to introduce small insertion-deletions (indels) through non-homologous end joining (NHEJ) [2]–[12] *in vivo* or targeted sequence changes by homology directed repair (HDR) *in vitro* [13], [14]. Compared with ZFNs, TALENs have generated considerable interest because of their simple and straightforward cipher code that guides DNA binding domain design, which is based on a 33–35 amino acid repetitive sequence [15], [16]. Each repeat encodes a single nucleotide binding specificity determined by the Repeat Variable Di-residues (RVDs) [15], [16]. TALENs can therefore be customized for targeting genomic sequence by assembling corresponding RVDs through highly developed methods [17]–[21].

TALENs have been used extensively in genome editing *in vitro* and *in vivo* in cells [18], [22], [23] and in many diverse species [24]–[27]. Recently, we reported a GoldyTALEN scaffold [28] with high efficiency as compared to other previously reported TALEN systems [25], [29]–[32] or ZFNs [8], [32] in zebrafish genome editing. GoldyTALENs were able to not only introduce small insertion-deletions (indels) [26], [28], but also enable new genome engineering approaches like targeted mutations via HDR [28].

The TALEN field has been implementing a range of systems and design approaches. In addition to related but divergent scaffolds, there are other common differences in TALEN designs, including the use of alternate-style RVDs, RVD composition, length of the DNA binding motif, as well as the spacer length between TALEN arms; these various guidelines for TALEN designs have been published previously based on other scaffolds or model systems [17], [20], [33]–[35]. One recent study suggests that several of these rules do not dictate TALEN efficiency in zebrafish, however, leaving the community with little direction when deciding on TALEN design [32]. Given these diverse protocols with highly variable activities, a simple yet flexible GoldyTALEN design with a high success rate for generating active reagents would be a valuable addition to the science community. Here, we report a simplified 15-RVD TALEN design with high *in vivo* genome targeting efficiency. We used these TALENs to engineer with high success targeted large deletions in this increasingly important animal model system, the zebrafish.

## Materials and Methods

### Zebrafish

All zebrafish work was completed under pre-approved animal care and use guidelines approved by the Mayo Clinic Institutional Animal Care and Use Committee.

### Design of GoldyTALENs

**Figure 1:**
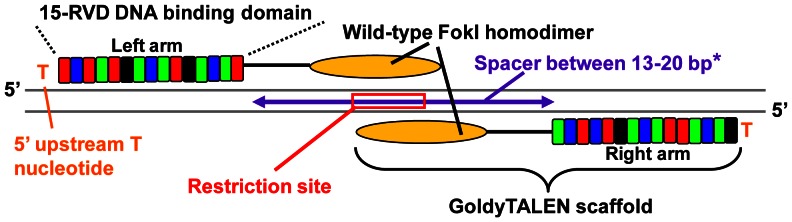
Design of 15-RVD GoldyTALENs. The design parameters of active 15-RVD GoldyTALEN pairs used in this study. Each TALEN arm consists of a DNA binding domain with 15-RVDs (14.5 TALE repeats), corresponding to 15-nucleotides DNA binding sequence proceeded by a 5′ T nucleotides and a 13 to 20 bp spacer in between 2 arms containing a restriction recognition sequence to assay activity. *Initial design parameter was spacer length between 11–20 bp and only the two inactive pairs, NPM1B P1 and P2 have spacers shorter than 13 bp.

All TALEN pairs were designed using Mojo Hand software (www.talendesign.org) [36], which is a freely accessible web-based tool to design TALENs with editable parameters and other features including the identification of a unique restriction site in the spacer sequence (Figure 1). Initial design parameters included 15-RVD (or 14.5 TALE repeats) TALENs targeting sites with a T nucleotide 5′ upstream of TALEN targeting sites and spacer lengths between 11–20 base pair (bp) with a unique restriction site close to the middle of the spacer sequence for screening of small indels induced through NHEJ. Modifications were made to these parameters later in the study as described. Specificity of TALENs was examined using NCBI Primer-BLAST (Table S4). All loci were amplified from wild-type fish and sequence determined to avoid any polymorphisms or mismatches within the TALEN binding site or the spacer.

### Synthesis of GoldyTALENs

**Figure 2:**
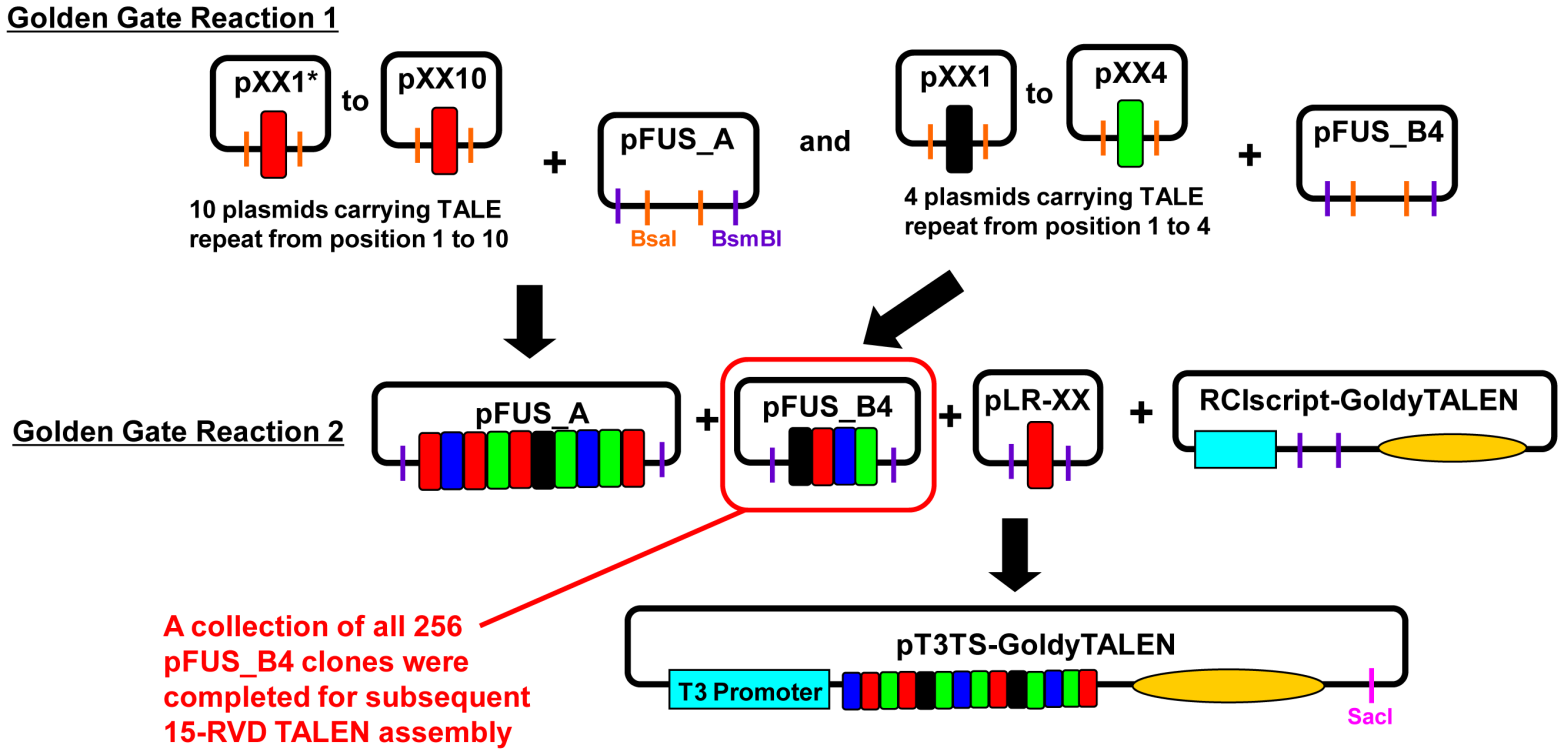
Synthesis of 15-RVD GoldyTALENs. A schematic diagram showing the assembly of 15-RVD GoldyTALENs by the Golden Gate method described earlier [17]. The RCIscript-GoldyTALEN backbone, all other component plasmids (as Golden Gate TALEN kit 2.0), and the 256 pFUS_B4 clones are distributed through Addgene. *XX denoted either NI, HD, NN or NG.

All TALEN constructs were synthesized with the Golden Gate method [17] using the GoldyTALEN scaffold [28] (Figure 2). The highly active RVD NN was used to target G nucleotide rather than the lower activity RVDs NK [34] or NH [35]. In the first Golden Gate reaction, intermediate constructs containing TALE repeats 1 to 10 and 11–14 were separately synthesized in pFUS_A and pFUS_B4 vectors, respectively. The two TALE repeat arrays from pFUS_A and pFUS_B4 as well as the last half-repeat carried in either pLR-NI, -HD, -NN or –NG were combined in the second Golden Gate reaction in the RCIscript-GoldyTALEN expression vector. GoldyTALEN constructs were subsequently linearized by SacI, and mRNA encoding each TALEN arm was *in vitro* transcribed using the mMESSAGE mMachine T3 Kit (Life Technologies, Grand Island, NY, USA).

### Injection of GoldyTALENs and screening for somatic mutations in zebrafish

The two mRNAs encoding each TALEN pair were injected into the cytoplasm of 1-cell stage wild-type zebrafish embryos, except the GFP(GM2) P1 TALEN pair that was injected into GFP transgenic line Tg(GBT309) [37]. Genomic DNA was extracted from both single and groups of 10 embryos at 48 hours-post-fertilization (hpf). Somatic small indels resulting from NHEJ were screened and quantified using a RFLP assay as previously described [28] (Table S5). To normalize variation between single embryos, TALEN activities were measured from genomic DNA extracted from a group of 10 embryos. Large deletions resulted from co-injecting 2 TALEN pairs were screened by PCR with primers flanking the 2 TALEN cutting sites (Table S5). Quantitative PCR (qPCR) with primers within the corresponding deleted fragments was used to estimate the percentage of large deletions in TALEN-injected embryos, with primers at an irrelevant locus used as an internal reference (Table S5). For all screening, only phenotypically normal embryos injected at the highest tolerated dose (>50% normal embryos at 48 hpf) (Table S6) were analyzed.

### Screening for germline transmitted deletions

Phenotypically normal injected embryos (same batch of those screened for somatic mutations) were raised and tail fins were biopsied after 8-weeks to screen for maintenance of induced mutations, as described [28]. Fish with positive fin-clip results were subsequently out-crossed with wild-type fish after 12-weeks, and F1 progeny were screened for germline mutations.

### Synthesis of pFUS_B4 collection

The complete collection of 256 pFUS_B4 (with all possible 4-RVD combination) used in the Golden Gate synthesis of 15-RVD TALENs was synthesized initially through a mix-reaction approach. Either NI, HD, NN or NG were added at position 1 and all 4-RVDs were added for position 2 to 4 in a single Golden Gate reaction to synthesized up to 256 possible pFUS_B4 clones in 4 reactions. Around 80% of possible combinations were identified through screening 192 colonies from each of the 4 reactions, and the remaining pFUS_B4 clones were synthesized individually

### Statistical analysis

The TALEN activities were calculated as the means of three separate experiments with the standard error of the means. To analyze the relationship between TALEN activity and weak RVDs (NI and NG) composition, the linear correlation coefficient was defined from the plot of individual TALEN activities against their percentages of weak RVDs as defined [35].

## Results

### Somatic screening showed a high success rate of the 15-RVD GoldyTALEN design

**Figure 3:**
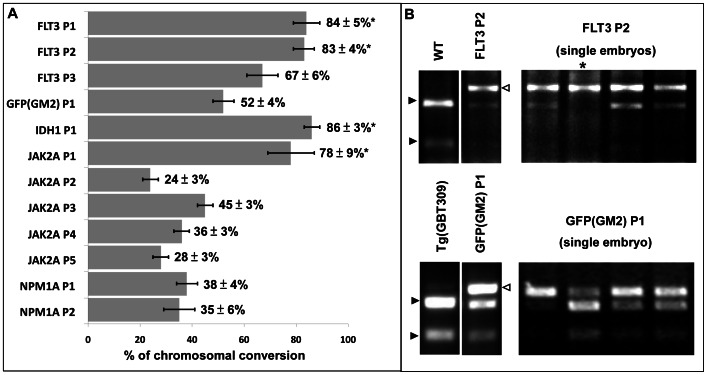
*In vivo* activity of 15-RVD GoldyTALENs. (A) *In vivo* activity (% of chromosome conversion at somatic level) of 15-RVD TALEN pairs. Results shown were averages of 3 separate experiments analyzing groups of 10 embryos. Error bars represent the standard error of the means. (B) Representative results of RFLP screening assay after injection of IDH1 P1 and GFP(GM2) P1. Open arrowheads indicate bands from completely digested WT PCR product and closed arrowheads represent uncut PCR product with small indels. *Marks single embryos with bi-allelic conversion.

**Figure 4:**
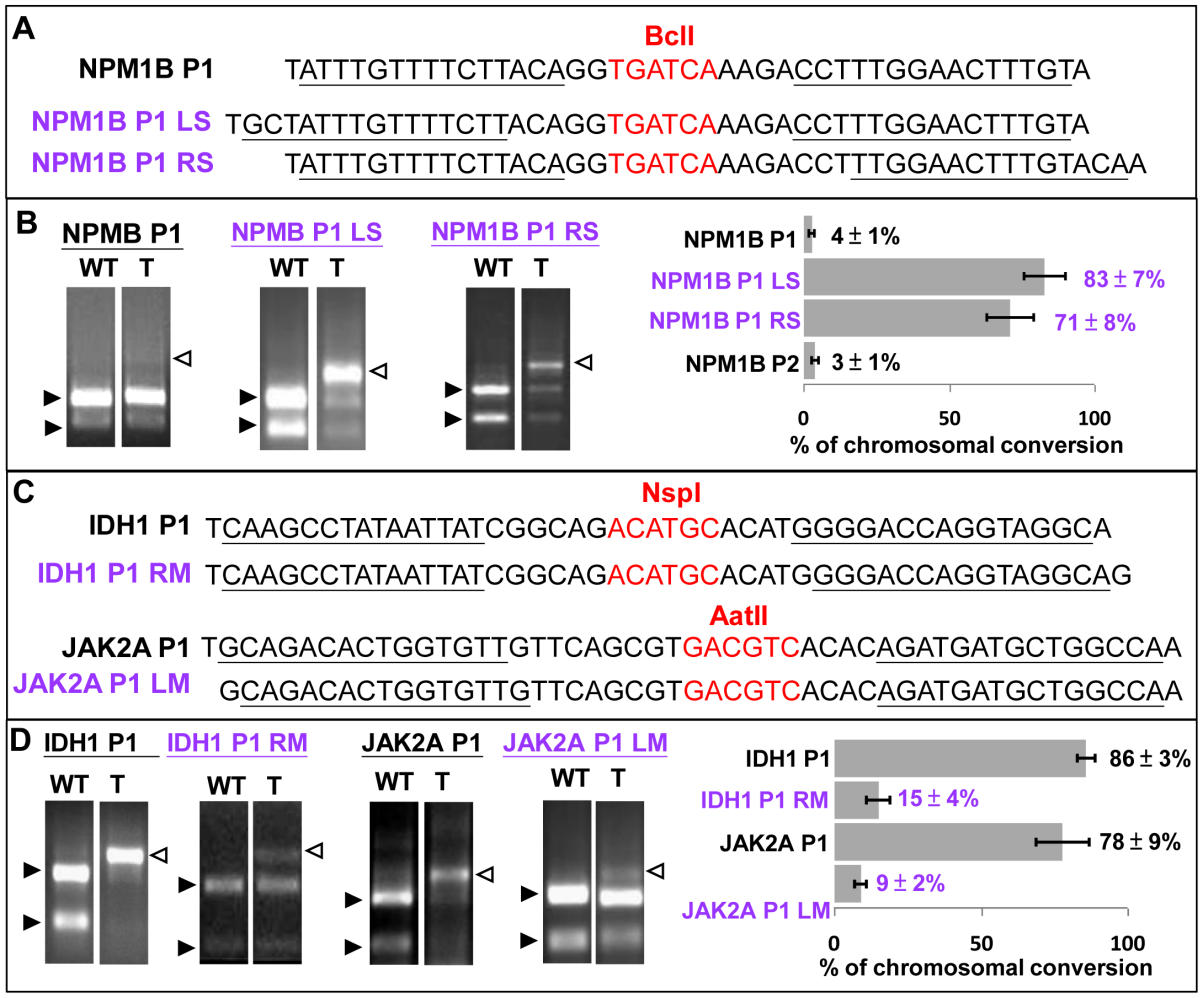
Importance of the spacer length and the 5′ T nucleotide in GoldyTALEN activity. (A) Modifications of NPMB1 P1 to LS and RS for longer spacers. (B) Both modified NPMBP1 TALEN pairs (NPM1B P1 LS and RS) showed significant increase in *in vivo* activity compared with the original shorter spacer design. (C) Modifications of IDH1 P1 and JAK2A P1 to remove the 5′ upstream T nucleotide at one of the TALEN arms. (D) Both modified TALEN pairs (IDH1 P1 RM and JAK2A P1 LM) showed significant reduction in *in vivo* activity compared with the original designs. Open arrowheads indicate bands from completely digested WT PCR product and closed arrowheads represent uncut PCR product with small indels. WT: wild-type; T: TALEN pair injected. Representative and average results of RFLP screening in 3 separate experiments analyzing group of 10 embryos are shown in the gel photo and graph, respectively. Error bars represent the standard error of the means.

TALENs are assembled as pairs for genome editing applications, with a wide range of lengths (9 to 30-RVD, including the final half repeat) for each arm reported to show activity [17]–[20], [35]. Notably, some TALENs as short as 15-RVDs (14.5 TALE repeats) have been shown to exhibit high activities [20], [27], consistent with the reported structural work demonstrating 15 bases of sequence recognition by TAL domains [38], [39]. Together, these data suggested that 15-RVD TALENs might be a simplified yet effective TALEN design approach. 15-RVD GoldyTALEN pairs (Figure 1) targeting nine loci in five zebrafish genes and GFP (Table S1) were designed based on these initial parameters (Figure 1 and see Methods section), and their *in vivo* activity was tested in zebrafish embryos. 11 out of 13 15-RVD GoldyTALEN pairs we tested showed efficient targeting, ranging from 24 to 84% chromosome conversion rate (Figure 3A and Figure S1). Two pairs targeting *npm1b* (NPM1B P1 and P2) showed significantly reduced activity compared to the other GoldyTALENs (Figure 4A and B). Bi-allelic chromosomal conversion, a hallmark of the GoldyTALEN system in zebrafish [28], was detected in 3 out of 13 TALEN pairs (Figure 3B).

### Spacer length and the 5′ T nucleotide are important for TALEN activity

One recent study proposed a hypothesis to distinguish and predict high efficacy from low efficacy TALENs by suggesting that RVDs could be sorted into ‘strong’ or ‘weak’ categories [35]. To better understand the basic requirements of an active GoldyTALEN pair, we correlated TALEN activity with the composition of strong (HD, NN) or weak (NI, NG) RVDs as well as the spacer length. No direct correlation between TALEN activity and the relative composition of different RVDs was noted (correlation coefficient = −0.149, Figure S2 and S3). However, NPM1B P1 and P2 with significantly reduced activity had a shorter spacer of 12 and 11 bp while all other successful GoldyTALENs were ≥13 bp (Table 1). To test if the spacer length is critical for TALEN activity, both left (NPM1B P1 LS) and right arms (NPM1B P1 RS) of NPM1B P1 were redesigned for a longer spacer (15 bp) while keeping a 5′ T nucleotide in place (Figure 4A). Shifting either side of the pair significantly restored the 15-RVD TALEN activity to 83±7 and 71±8% (Figure 4B). Furthermore, the 5′ T nucleotide found upstream of most active TALEN binding sites was tested for its importance in GoldyTALEN activity. The right and left arms of IDH1 P1 and JAK2A P1, respectively, were shifted one base to remove the 5′ upstream T nucleotide (Figure 4C). Interestingly, the activity of these modified TALEN pairs (IDH1 P1 RM and JAK2A P1 LM) was greatly reduced to 15±4 and 9±2% (Figure 4D), unambiguously demonstrating an important functional role for this base in TALE DNA binding.

**Table 1:** Correlation of spacer lengths and somatic activities of 15-RVD GoldyTALENs.

TALEN Pair*	Spacer Length (bp)	Somatic Activities (% chromosomal conversion)
IDH1 P1	16	86±3%*
FLT3 P1	16	84±5%*
FLT3 P2	15	83±4%*
NPM1B P1 LN	15	83±7%*
JAK2A P1	19	78±9%
NPM1B P1 RN	15	71±8%
FLT3 P3	14	67±6%
GFP(GM2) P1	19	52±4%
JAK2A P3	18	45±3%
NPM1A P1	13	38±4%
JAK2A P4	15	36±3%
NPM1A P2	18	35±6%
JAK2A P5	19	28±3%
JAK2A P2	16	24±3%
IDH1 P1 RM†	17	15±4%
JAK2A P1 LM†	18	9±3%
NPM1B P2	11	3±1%
NPM1B P1	12	4±1%

* shown in decreasing order of activity.

† modified without the 5′ T nucleotide at one of the TALEN arms.

### Targeted deletion could be introduced by co-injecting two pairs of TALEN

**Figure 5:**
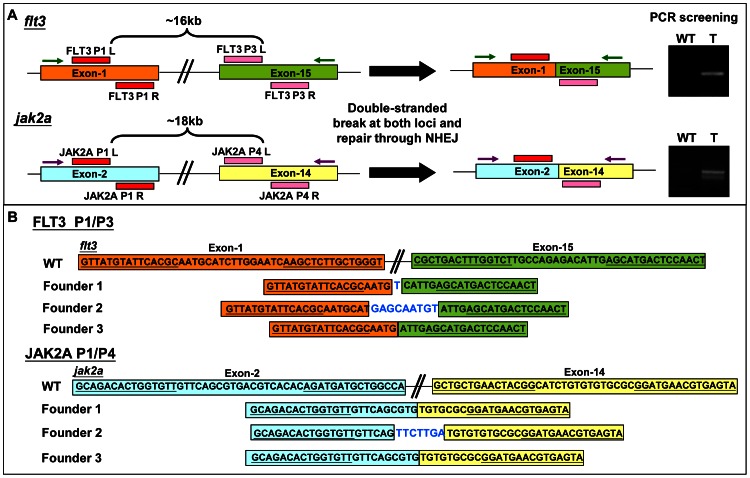
Deletion of large genomic fragments with two pairs of TALEN. (A) Schematic diagram showing the strategy of introducing large genomic deletions at *flt3* and *jak2a* loci using two TALEN pairs. Green and purple arrows represent primer pairs used for PCR screening of corresponding large deletion. WT: wild-type; T: TALEN pairs injected. (B) Sequences of germline-transmitted large deletions in F1 embryos. Sequence of a single mutant F1 embryo from each founder is shown. Underlined are TALEN binding sites. Sequences in blue represent insertions.

Genetic nulls are classically defined as physical deficiencies in a particular locus. The high activity of GoldyTALENs and the early data from TALEN work using the silkworm [40] suggested that injecting two pairs of TALENs could be a viable approach to specifically delete a large contiguous region of the genome. To test whether 15-RVD GoldyTALENs would work in this capacity in zebrafish, either FLT3 P1 and P3 or JAK2A P1 and P4 TALEN pairs were injected into zebrafish embryos, an approach expected to delete fragments sized around 16 kilobases (kb) and 18 kb, respectively (Figure 5). PCR screening indicated a high penetrance of the expected large deletions by both TALEN combinations at the somatic cell level, detected in 81±11% (FLT3 P1/P3) and 84±9% (JAK2A P1/P4) of injected embryos. The percentage of deletion in injected embryos was 27±3% (FLT3 P1/P3) and 19±4% (JAK2A P1/P4) as estimated by qPCR.

### Mutations introduced by 15-RVD TALENs are inheritable

To test if the small indels and large deletions induced by 15-RVD TALEN pairs were inheritable, embryos injected with FLT3 P2, NPM1B P1 LS, NPM1A P1 or NPM1A P2 as well as embryos co-injected with FLT3 P1/P3 or JAK2A P1/P4 were raised to sexual maturity. Injected fish with stable mutations in DNA from adult tail tissue were subsequently out-crossed and their progeny were screened for germline-transmitted mutations. Encouragingly, expected mutations from all six batches were transmitted to the germline at a high frequency. 18 to 100% of screened F0 embryos injected with a single GoldyTALEN pair carry germline mutations of small indels whereas 28 and 31% of screened F0 embryos carry germline mutations of large deletions, respectively, at *flt3* and *jak2a* loci. (Figure 5B, Table S2 and S3).

## Discussion

Recent advances in TALEN technology have shaped this custom restriction enzyme system into highly useful genome engineering tools. Designing an efficient TALEN pair is perhaps the most crucial step for successful genome engineering. The extensive nature of the reported guidelines have large variations in the description of critical details for achieving working activity with different TALEN systems suggesting that parameters controlling TALEN activity may be scaffold-specific. Given the high activity of the GoldyTALEN system described to date, we investigated flexibility and other constraints in the GoldyTALEN design. A 15-RVD design was initially chosen because 15 bp at both arms offered enough DNA binding specificity *in silico* (Table S4), and the GoldyTALENs targeting *moesina* with a 15-RVD right arm showed high activity in our previous study [28]. Recent studies also indicated that 15-RVD DNA targeting site is the minimum length for an effective TALEN [20] with minimal off-target effects [35]. In this study, we chose to use RVD NN to target all G residues because studies suggested that though NK or NH has higher specificity towards G residue, TALE domains with NN showed highest activity [34], [35]. Further study is needed to draw conclusions comparing the efficiency of NN and NH in the GoldyTALEN scaffold.

Initial screening data showed a high success rate with 12 out of 14 TALEN pairs showing somatic chromosomal conversion activity beyond 19%; the only 2 exceptional pairs were NPM1B P1 and P2. Since these TALENs had the shortest spacers (12 and 11 bp) among the cohort, we investigated if we could restore their activity by simply modifying the spacer. Extending the spacer of NPM1B P1 to 15 bp by shifting either left or right arm 3 bp while keeping other variables constant significantly restored the activity indicating that 15-RVD TALEN pairs need at least a 13 bp spacer. On the other hand, there is no clear correlation between TALEN activity and RVD compositions. Although both NPM1B P1 and P2 had a relatively high percentage of proposed ‘weak’ RVD (NI and NG), the high activity of NPM1B P1 LS and RS, sharing a similar RVD composition with NPM1B P1, suggests that spacer length is more important than RVD composition. In addition, to determine if the 5′ T nucleotide is necessary for TALEN activity, IDH1 P1 and JAK2A P1 were modified such that one of the TALEN arms did not follow a T nucleotide. Interestingly, removal of the T nucleotide at only one arm greatly reduced TALEN activity, highlighting the importance of the preceding nucleotide at least in the 15-RVD GoldyTALEN design.

The ability to obtain engineered modifications within the germline is crucial for genome editing. Consistent with our previous study with GoldyTALENs [28], small indels introduced by 15-RVD GoldyTALENs with relatively high (FLT3 P2, NPMB1 LS) or low (NPM1A P1, NPM1A P2) activities were transmitted to the germline at high frequencies. A larger genomic deletion could be introduced *in vivo* using two pairs of TALENs in the silkworm [40], and we investigated if these highly active 15-RVD GoldyTALENs could serve the same purpose. At both loci tested, large deletions up to 18 kb were successfully introduced and also transmitted through the germline with high efficiencies (13–58% transmission rate from individual founder fish to F1 offspring). This strategy could be employed to generate null zebrafish mutations at critical loci. Recently, Gupta et al [41] reported the deletion of large genomic sequences in zebrafish using a similar strategy. Their deletion frequency in somatic tissue of the F0 embryos and germline transmission rates ranged from 0.7–15% and 2–13%, respectively, which are lower than the results reported here. While deletion size differences and locus-specific effects may also contribute to the observed efficacy differences, we believe the higher efficiencies we noted may also result from higher activity of individual GoldyTALENs. The GoldyTALENs we used to generate large genomic deletions had activity ranging from 45–84% compared to the 15–61% activity rates reported by Gupta et al. Overall, the 15-RVD GoldyTALENs with spacers ranging from 13–19 bp represent an active design with high success rate. The only additional substantive design restriction is the requirement of the 5′T nucleotide upstream. This design can be easily employed by changing the default parameters of our TALEN design software, Mojo Hand (www.talendesign.org) [36]. The average number of TALEN targeting sites identified with unique restriction sites in the spacer reached 1513 per 1 kb of genomic fragments in the target loci used here (including overlapping binding sites), and the number still reached 216 per 1 kb even if we further restricted the spacer length to 15–17 bp. Given that the restriction recognition site in the spacer is not an absolute requirement with the development of melting curve analysis [42], the flexibility of this design enables efficient targeting almost anywhere in the genome.

**Figure 6:**
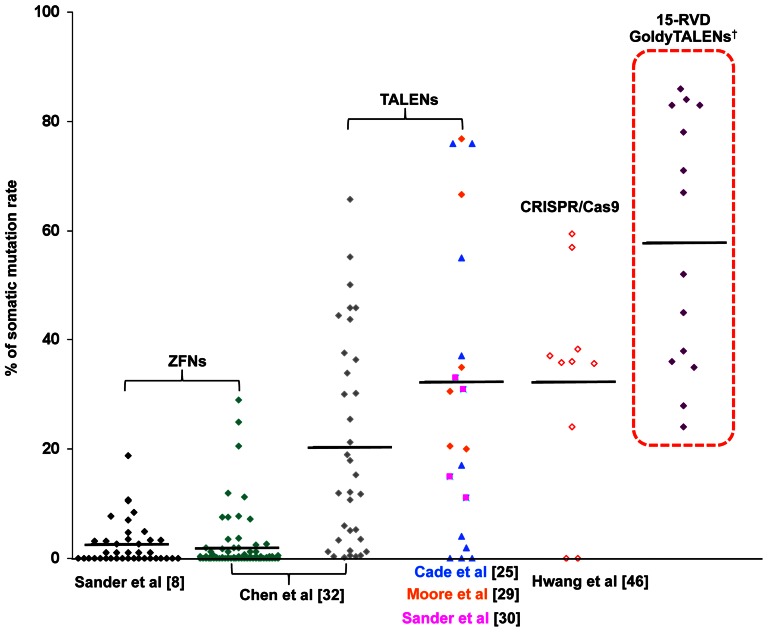
Somatic efficacy of 15-RVD TALEN compared with other reported TALEN scaffolds, ZFN and CRISPR/Cas9. Plot of somatic mutation rates in zebrafish genome editing using ZFNs [8], [32], TALENs [25], [29], [30], [32] and CRISPR/Cas9 [46] from representative publications together with 15-RVD TALENs from this study. *Only Homo-dimeric TALENs were compared. ^†^Only 15-RVD TALENs with spacer lengths between 13–20 bp and a T nucleotide preceding the TALEN binding site were included.

Recently, the CRISPR-associated (Cas) (CRISPR/cas9) [43]–[45] system was reported to successfully introduce somatic indels in zebrafish [46], [47]. This RNA-guided system offers the advantage of easy assembly and the possibility of simultaneously introducing multiple mutations at the same time. Nevertheless, the present simple and flexible 15-RVD GoldyTALEN design (with spacer between 13–20 bp and T nucleotide preceding both TALEN binding site) offers noticeably higher somatic efficacy (averaging 58±6%) and overall success rates using the updated design parameters (100%) (Figure 6). The requirement of TALENs to work in pairs may also offer higher specificity compared with the 12–20 recognition nucleotides in the single-guide RNAs [44]. Finally, the reported 15-RVD TALEN assembly by the Golden Gate method is based on the 10 (pFUS_A)+4 (pFUS_B4)+1 (p-LR) architecture [17]. The 15-RVD design constraint offers a modified design approach whereby all possible pFUS_B clones could be pre-assembled. To accomplish this, each of the possible 256 pFUS_B4 clones were assembled into a single collection to facilitate future 15-RVD TALEN construction that is fully backward-compatible with the popular Golden Gate assembly platform of Cermak et al [17] and available through Addgene.

## Supporting Information

Figure S1 *In vivo* activity of 15-RVD GoldyTALENs. Representative results of RFLP screening assay of all other 15-RVD GoldyTALENs tested. Open arrow heads indicate bands from completely digested WT PCR product and closed arrowheads represent uncut PCR product with small indels. *An extra restriction site appears in PCR product outside the spacer resulted in a 3 bands pattern in WT embryos.
(DOC)

Figure S2 RVD composition of 15-RVD GoldyTALENs. The RVD sequences of GoldyTALEN pairs and the percentage of predicted weak RVDs (NI, NG) [35]. *TALEN pairs are shown in order of decreasing *in vivo* activity.
(DOC)

Figure S3 Correlation between weak RVDs composition and somatic activity of 15-RVD GoldyTALENs. Scatter plot of GoldyTALENs activities against their percentages of weak RVD modules with the linear regression line. R^2^ = determination coefficient and R = correlation coefficient.
(DOC)

Table S1 15-RVD GoldyTALENs used in this study.
(DOC)

Table S2 Fin-clip and germline transmission rates of mutations introduced by 15-RVD GoldyTALENs.
(DOC)

Table S3 Sequences of germline transmitted small indels induced by 15-RVD GoldyTALENs in F1 embryos.
(DOC)

Table S4 Specificity of 15-RVD GoldyTALENs.
(DOC)

Table S5 Primer sequences used in this study.
(DOC)

Table S6 Highest tolerable injection dosage of TALEN mRNA.
(DOC)
