# Supplementary material for: High Efficiency In Vivo Genome Engineering with a Simplified 15-RVD GoldyTALEN Design
Source: PLoS One. 2013 May 29;8(5):e65259. doi: 10.1371/journal.pone.0065259 (PMC3667041; doi:10.1371/journal.pone.0065259)
Supplement: Figure S1 — In vivo activity of 15-RVD GoldyTALENs. Representative results of RFLP screening assay of all other 15-RVD GoldyTALENs tested. Open arrow heads indicate bands from completely digested WT PCR product and closed arrowheads represent uncut PCR product with small indels. *An extra restriction site appears in PCR product outside the spacer resulted in a 3 bands pattern in WT embryos. (DOC) [file pone.0065259.s001.doc]

**FLT3 P3**

**FLT3 P1**

**JAK2A P2**

**JAK2A P4**

**JAK2A P3**

**JAK2A P5**

**NPM1A P2**

**NPM1A P1**

**NPM1B P2**


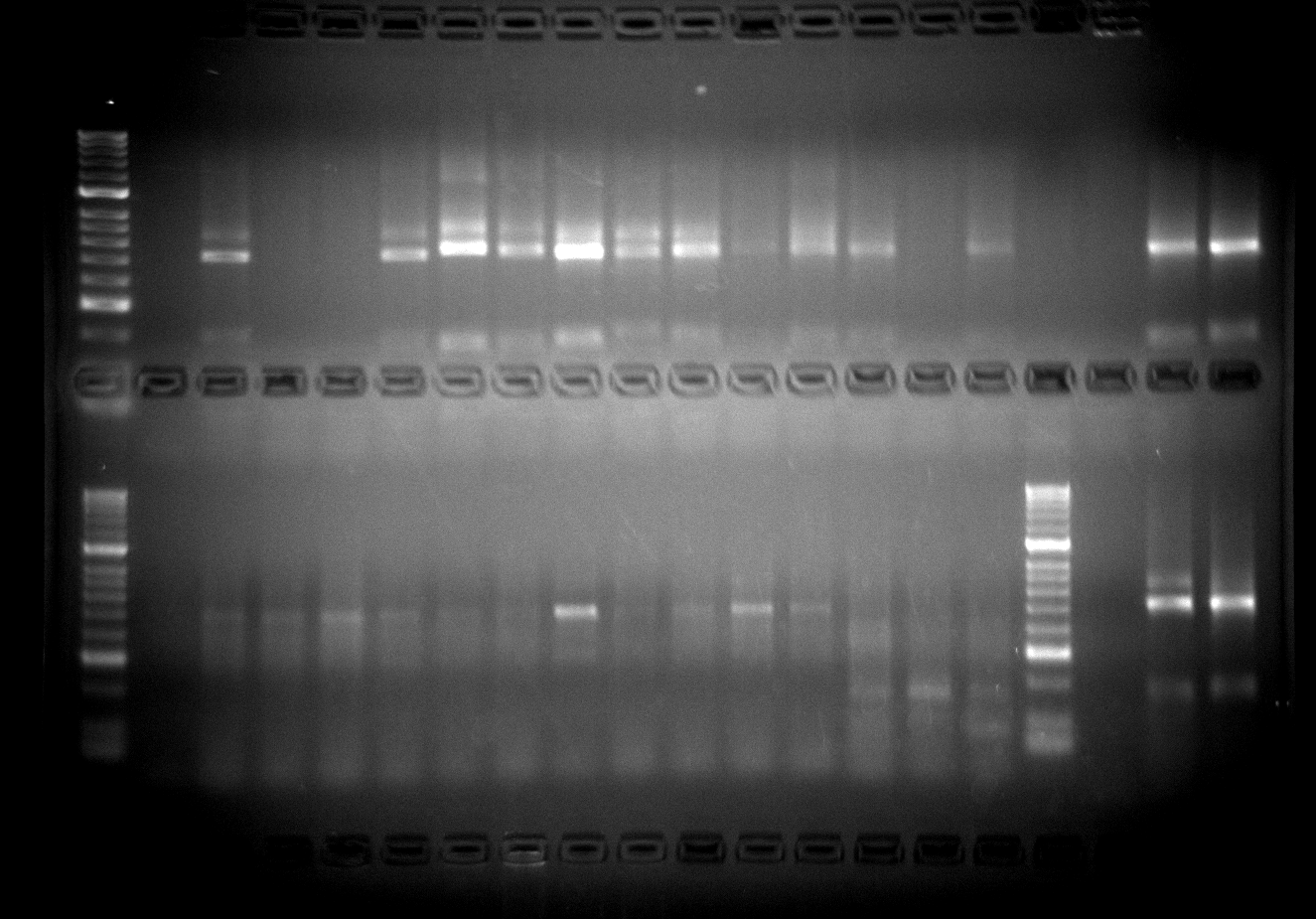

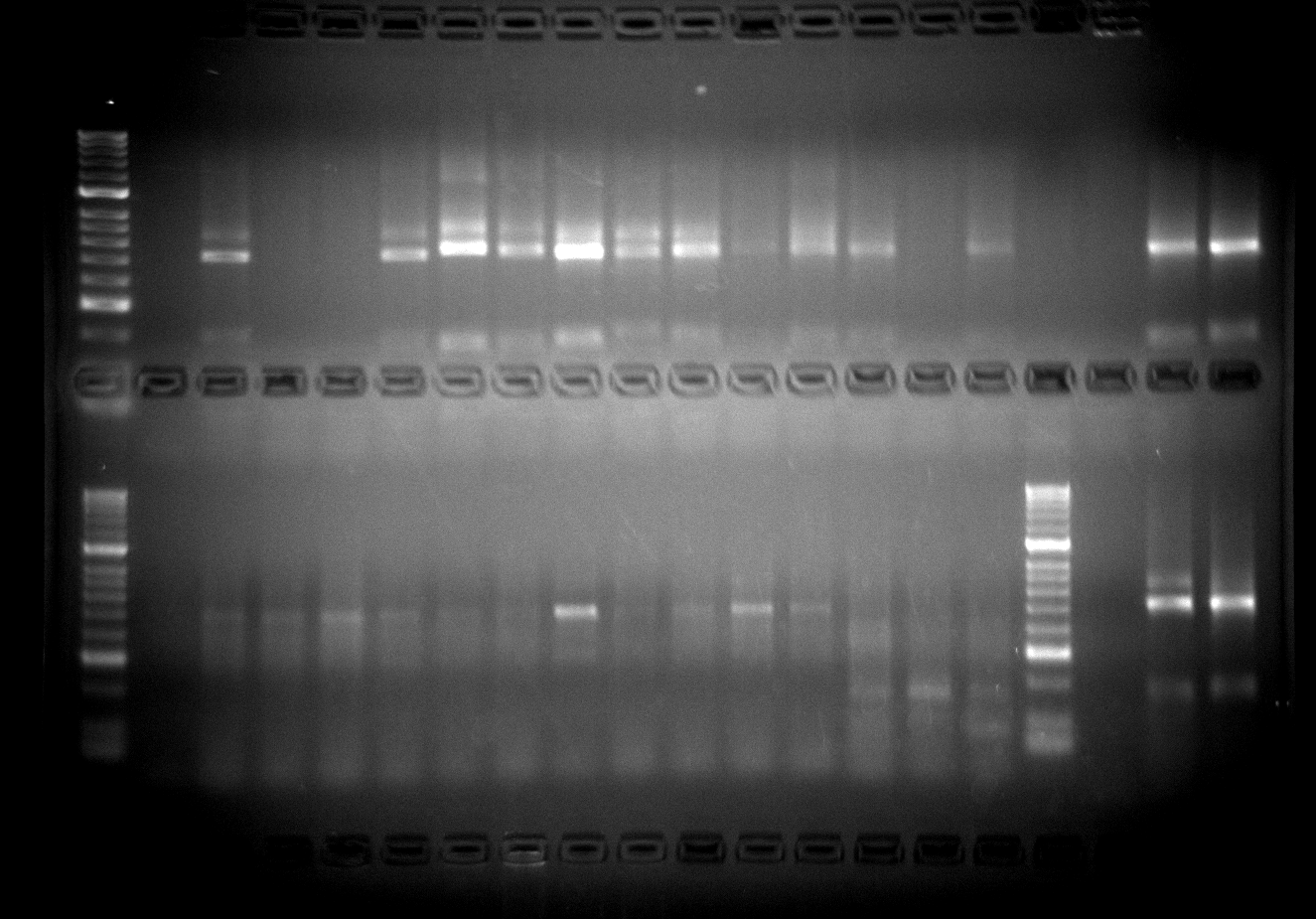

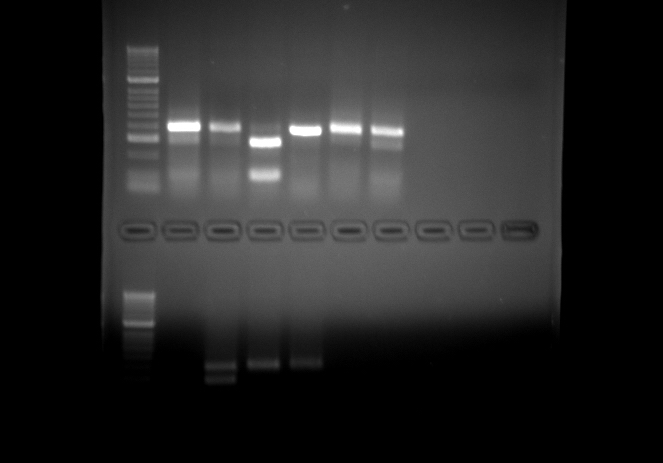

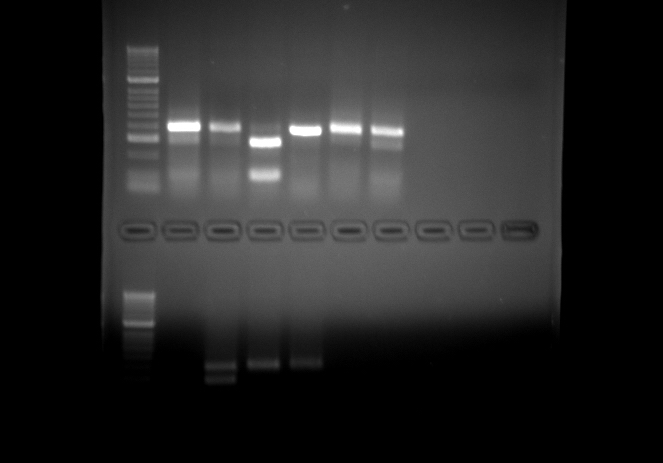

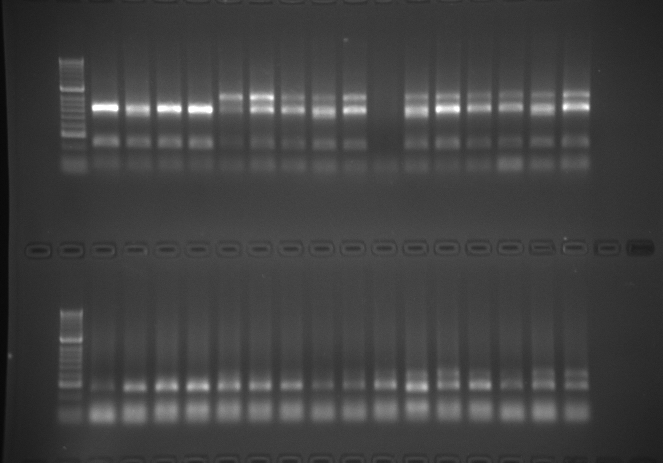

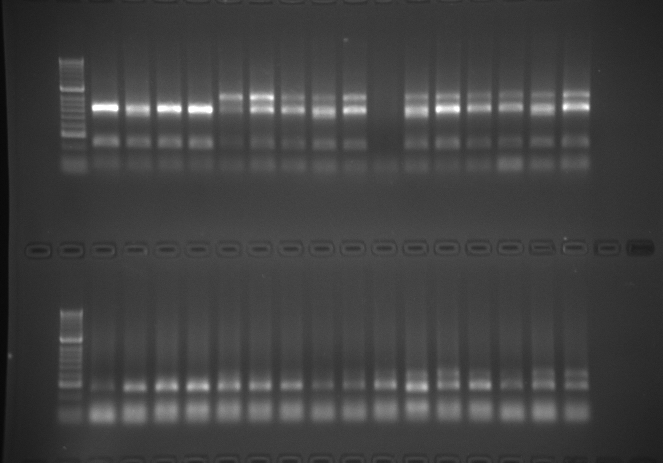

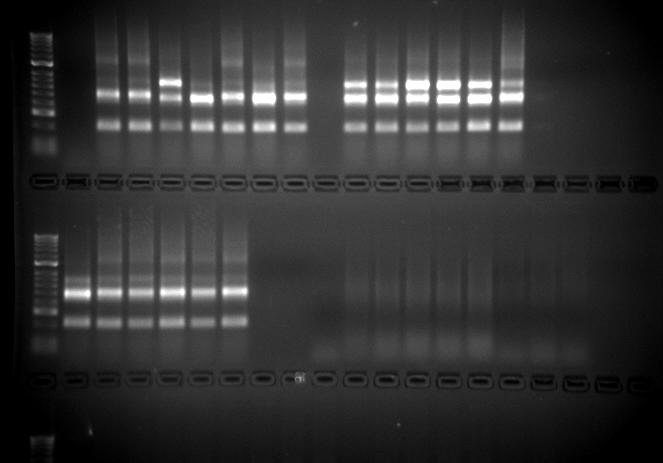

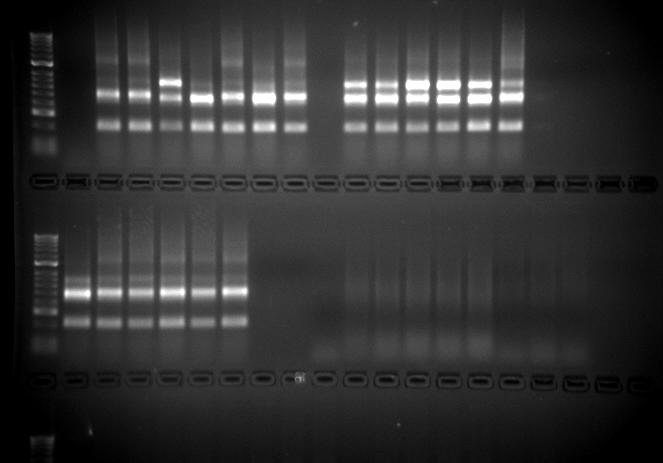

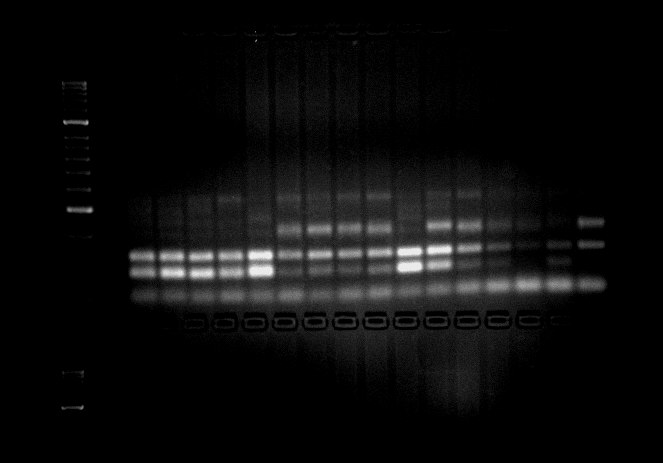

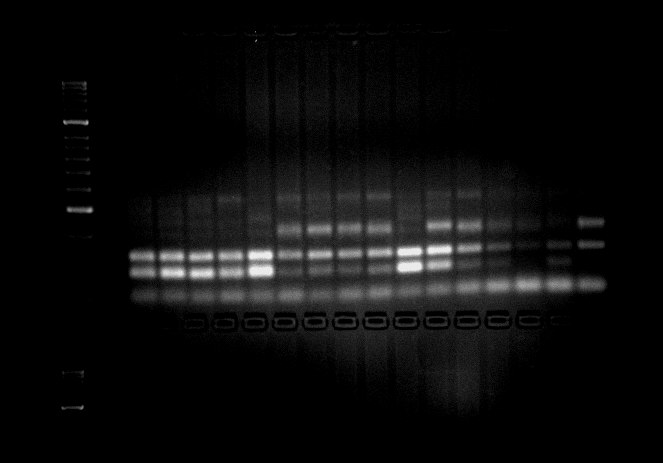

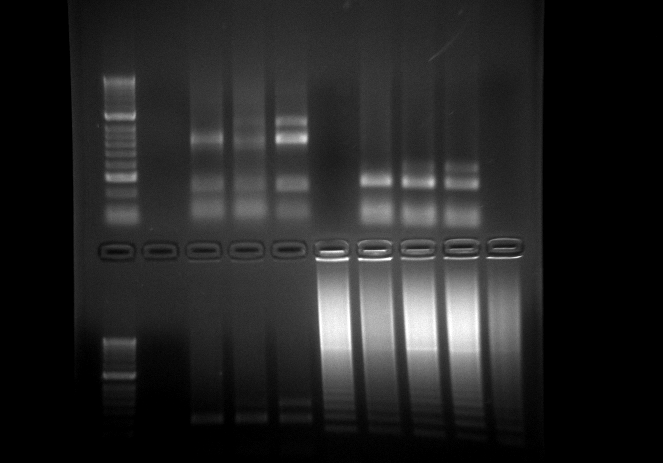

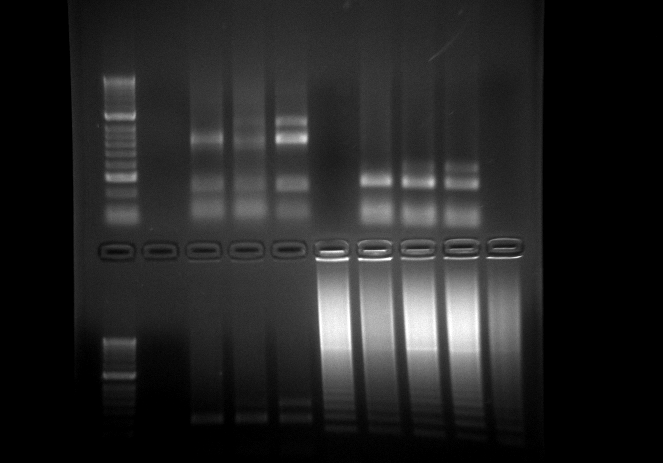

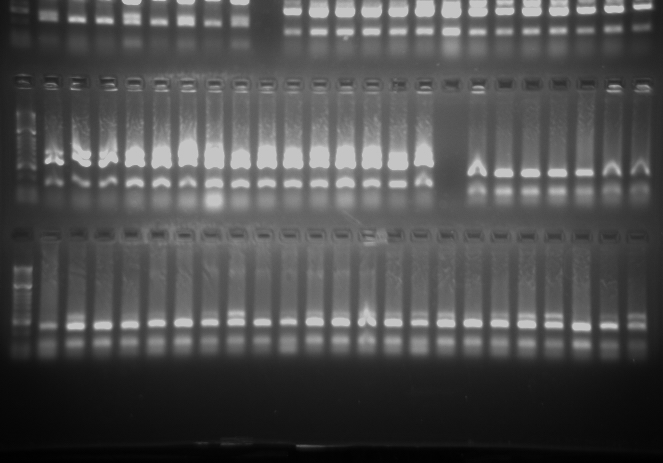

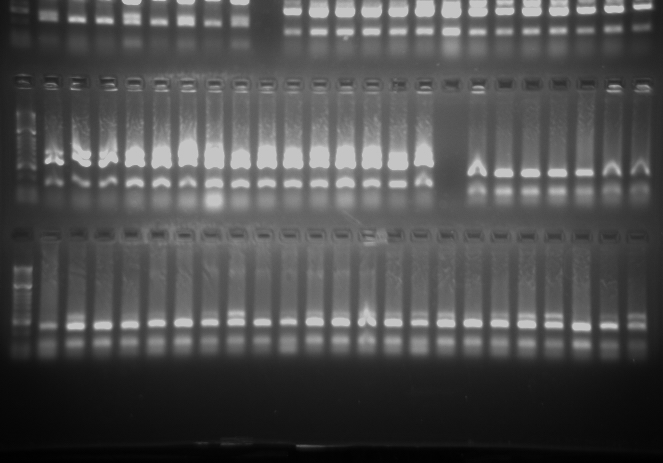

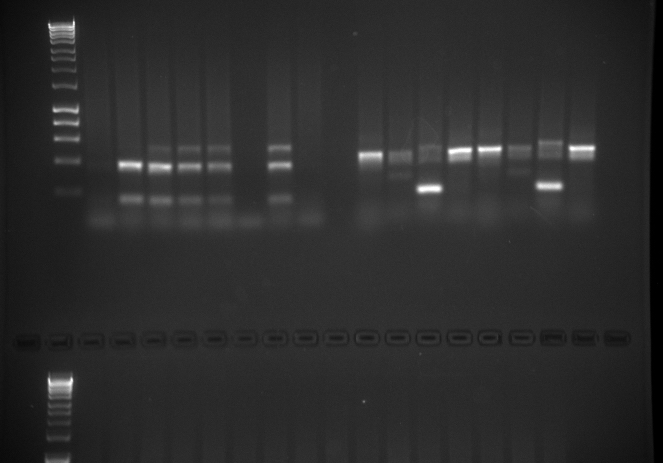

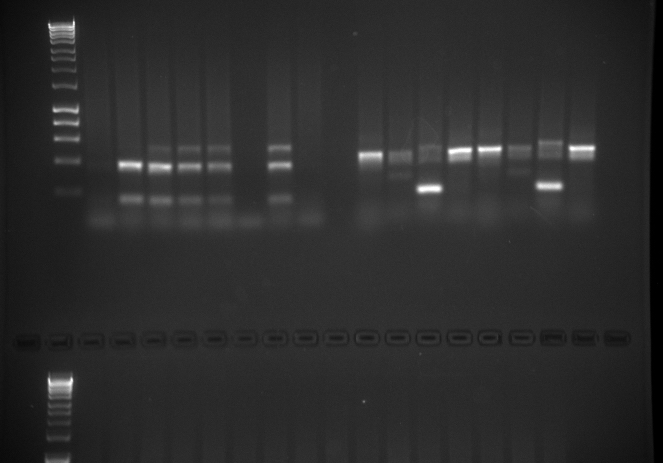

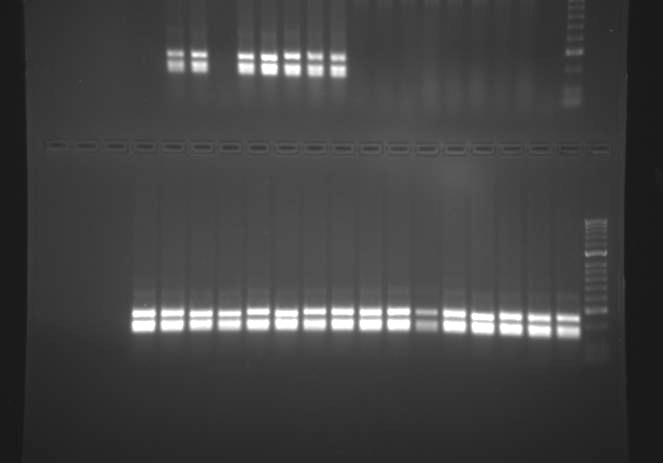

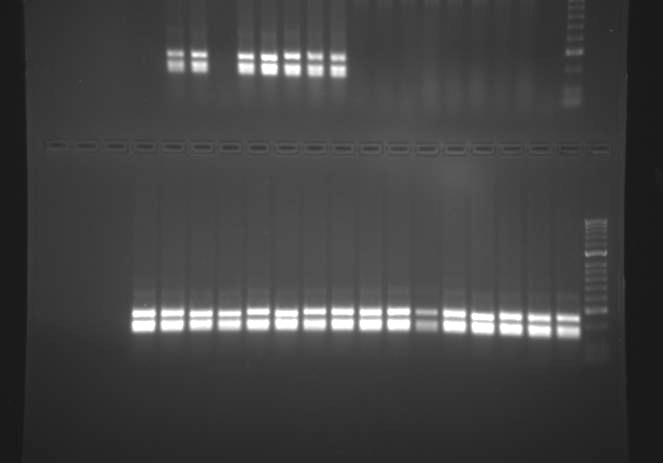


WT T

WT T

WT T

WT T

WT T

WT* T

WT T

WT T

WT* T

**Supplementary Figure S1. *In vivo* activity of 15-RVD GoldyTALENs.** Representative results of RFLP screening assay of all other 15-RVD GoldyTALENs tested. Open arrow heads indicate bands from completely digested WT PCR product and closed arrowheads represent uncut PCR product with small indels. *****An extra restriction site appears in PCR product outside the spacer resulted in a 3 bands pattern in WT embryos.
