# Supplementary material for: High Efficiency In Vivo Genome Engineering with a Simplified 15-RVD GoldyTALEN Design
Source: PLoS One. 2013 May 29;8(5):e65259. doi: 10.1371/journal.pone.0065259 (PMC3667041; doi:10.1371/journal.pone.0065259)
Supplement: Figure S2 — RVD composition of 15-RVD GoldyTALENs. The RVD sequences of GoldyTALEN pairs and the percentage of predicted weak RVDs (NI, NG) [35]. *TALEN pairs are shown in order of decreasing in vivo activity. (DOC) [file pone.0065259.s002.doc]

**Supplementary Figure S2. RVD composition of 15-RVD GoldyTALENs.** The RVD sequences of GoldyTALEN pairs and the percentage of predicted weak RVDs (NI, NG) [35]. *TALEN pairs are shown in order of decreasing *in vivo* activity.
