# Supplementary material for: High Efficiency In Vivo Genome Engineering with a Simplified 15-RVD GoldyTALEN Design
Source: PLoS One. 2013 May 29;8(5):e65259. doi: 10.1371/journal.pone.0065259 (PMC3667041; doi:10.1371/journal.pone.0065259)
Supplement: Figure S3 — Correlation between weak RVDs composition and somatic activity of 15-RVD GoldyTALENs. Scatter plot of GoldyTALENs activities against their percentages of weak RVD modules with the linear regression line. R2 = determination coefficient and R = correlation coefficient. (DOC) [file pone.0065259.s003.doc]

**Supplementary Figure S3. Correlation between weak RVDs composition and somatic activity of 15-RVD GoldyTALENs.** Scatter plot of GoldyTALENs activities against their percentages of weak RVD modules with the linear regression line. R^2^ = determination coefficient and R = correlation coefficient.
