# Supplementary material for: High Efficiency In Vivo Genome Engineering with a Simplified 15-RVD GoldyTALEN Design
Source: PLoS One. 2013 May 29;8(5):e65259. doi: 10.1371/journal.pone.0065259 (PMC3667041; doi:10.1371/journal.pone.0065259)
Supplement: Table S1 — 15-RVD GoldyTALENs used in this study. (DOC) [file pone.0065259.s004.doc]

**Supplementary Table S1. 15-RVD GoldyTALENs used in this study**

| **TALEN Pair** | **Targeting Locus** | **TALEN binding and spacer sequence*** | **Restriction enzyme^†^** |
| --- | --- | --- | --- |
| FLT3 P1 | *flt3* exon-1 | TGTTATGTATTCACGCAATGCATCTTGGAATCAAGCTCTTGCTGGGTA | NsiI |
| FLT3 P2 | *flt3* exon-2 | TTAGTGTGCACTTCTGAGGGGTACCCAAAACCAACTCTCAGGTGGTA | KpnI |
| FLT3 P3 | *flt3* exon-15 | TCGCTGACTTTGGTCTTGCCAGAGACATTGAGCATGACTCCAACTA | BcoDI |
| GFP(GM2) P1 | *GFP* (GM2-variant) | TACTGGAAAACTACCTGTTCCATGGCCAACACTTGTCACTACTCTCGCGTA | MscI |
| IDH1 P1 | *idh1* exon-3 | TCAAGCCTATAATTATCGGCAGACATGCACATGGGGACCAGGTAGGCA | NspI |
| IDH1 P1 RM | *idh1* exon-3 | TCAAGCCTATAATTATCGGCAGACATGCACATGGGGACCAGGTAGGCAG | NspI |
| JAK2A P1 | *jak2a* exon-2 | TGCAGACACTGGTGTTGTTCAGCGTGACGTCACACAGATGATGCTGGCCAA | AatII |
| JAK2A P1 LM | *jak2a* exon-2 | GCAGACACTGGTGTTGTTCAGCGTGACGTCACACAGATGATGCTGGCCAA | AatII |
| JAK2A P2 | *jak2a* exon-2 | TGTTGTTCAGCGTGACGTCACACAGATGATGCTGGCCAACACAGAGAA | MslI |
| JAK2A P3 | *jak2a* intron-2 | TCAGTGTTGTGTGACCTGTGTGTGTGAGCGGGAGCAGATGAGTGTGTACA | BsrBI |
| JAK2A P4 | *jak2a* exon-14 | TGCTGCTGAACTACGGCATCTGTGTGTGCGCGGATGAACGTGAGTAA | MslI |
| JAK2A P5 | *jak2a* exon-14 | TACCTGCTGCTGAACTACGGCATCTGTGTGTGCGCGGATGAACGTGAGTAA | SfaNI |
| NPM1A P1 | *npm1a* exon-11 | TTCCCAAGGTTGTTGAGGAGCTCTGGAAGTGGAGACAGACTGTCA | SacI |
| NPM1A P2 | *npm1a* exon-11 | TTGTTGAGGAGCTCTGGAAGTGGAGACAGACTGTCAAATAAATGA | BcoDI |
| NPM1B P1 | *npm1b* exon-11 | TATTTGTTTTCTTACAGGTGATCAAAGACCTTTGGAACTTTGTA | BclI |
| NPM1B P1 LS | *npm1b* exon-11 | TGCTATTTGTTTTCTTACAGGTGATCAAAGACCTTTGGAACTTTGTA | BclI |
| NPM1B P1 RS | *npm1b* exon-11 | TATTTGTTTTCTTACAGGTGATCAAAGACCTTTGGAACTTTGTACAA | BclI |
| NPM1B P2 | *npm1b* exon-11 | TGATCAAAGACCTTTGGAACTTTGTACAATCACTAAAAAAGTA | BsrGI |

*Underlined is the TALEN binding site with only the (+) strand shown (the right arm of each TALEN pair binds to the reverse complementary strand). Boxed text shows the endogenous genomic restriction enzyme recognition sequence nearby the TALEN cut site.

**^†^**Restriction enzyme used for screening somatic TALEN activity.
