# Supplementary material for: High Efficiency In Vivo Genome Engineering with a Simplified 15-RVD GoldyTALEN Design
Source: PLoS One. 2013 May 29;8(5):e65259. doi: 10.1371/journal.pone.0065259 (PMC3667041; doi:10.1371/journal.pone.0065259)
Supplement: Table S2 — Fin-clip and germline transmission rates of mutations introduced by 15-RVD GoldyTALENs. (DOC) [file pone.0065259.s005.doc]

**Supplementary Table S2. Fin-clip and germline transmission rates of mutations introduced by 15-RVD GoldyTALENs**

| **TALEN Pair(s)** | **# of Tail Fin Biopsy Positive F0** | **# of Founder F0*** | **Germline transmission rate^†^** | **# of mutant embryos in F1** | |
| --- | --- | --- | --- | --- | --- |
| **FLT3 P2** | 5 (7 screened) | 2 (2 screened) | 71% | Founder 1 | 6 (8 screened) |
|  |  |  |  | Founder 2 | 3 (8 screened) |
|  |  |  |  |  |  |
| **NPM1B P1 LS** | 8 (8 screened) | 2 (2 screened) | 100% | Founder 1 | 2 (13 screened) |
|  |  |  |  | Founder 2 | 3 (12 screened) |
|  |  |  |  |  |  |
| **NPM1A P1** | 5 (7 screened) | 2 (4 screened) | 36% | Founder 1 | 4 (20 screened) |
|  |  |  |  | Founder 2 | 3 (16 screened) |
|  |  |  |  |  |  |
| **NPM1A P2** | 5 (11 screened) | 2 (5 screened) | 18% | Founder 1 | 1 (16 screened) |
|  |  |  |  | Founder 2 | 3 (16 screened) |
|  |  |  |  |  |  |
| **FLT3 P1/P3** | 11 (15 screened) | 3 (7 screened) | 31% | Founder 1 | 4 (10 screened) |
|  |  |  |  | Founder 2 | 7 (12 screened) |
|  |  |  |  | Founder 3 | 2 ( 8 screened) |
|  |  |  |  |  |  |
| **JAK2A P1/P4** | 15 (23 screened) | 3 (7 screened) | 28% | Founder 1 | 4 (16 screened) |
|  |  |  |  | Founder 2 | 3 (16 screened) |
|  |  |  |  | Founder 3 | 2 (16 screened) |

*Out of tail fin biopsy positive F0 embryos.

**^†^**Percentage of fin-clip positive multiplied by the percentage of F0 screened with germline transmission.
