# Supplementary material for: High Efficiency In Vivo Genome Engineering with a Simplified 15-RVD GoldyTALEN Design
Source: PLoS One. 2013 May 29;8(5):e65259. doi: 10.1371/journal.pone.0065259 (PMC3667041; doi:10.1371/journal.pone.0065259)
Supplement: Table S3 — Sequences of germline transmitted small indels induced by 15-RVD GoldyTALENs in F1 embryos. (DOC) [file pone.0065259.s006.doc]

**Supplementary Table S3. Sequences of germline transmitted small indels induced by 15-RVD GoldyTALENs in F1 embryos.**

| **TALEN Pair** |  | **Sequence of targeting locus in F1 mutant** |
| --- | --- | --- |
| **FLT3 P2** | Wild-type | TAGTGTGCACTTCTGAGGGGTACCCAAAACCAACTCTCAGGTGGT |
|  | Founder 1 | TAGTGTGCACTTCT.....TCT.....AACCAACTCTCAGGTGGT |
|  | Founder 2 | TAGTGTGCACTTCTGA...........AACCAACTCTCAGGTGGT |
| **NPM1B LS** | Wild-type | GCTATTTGTTTTCTTACAGGTGATCAAAGACCTTTGGAACTTTGT |
|  | Founder 1 | GCTATTTGTTTTCTT**·········**CAAAGACCTTTGGAACTTTGT |
|  | Founder 2 | GCTATTTGTTTTCTTACAGG**····**CAAAGACCTTTGGAACTTTGT |
| **NPM1A P1** | Wild-type | TCCCAAGGTTGTTGAGGAGCTCTGGAAGTGGAGACAGACTGTC |
|  | Founder 1 | TCCCAAGGTTGTTGAGG**·······**GAAGTGGAGACAGACTGTC |
|  | Founder 2 | TCCCAAGGTTGTTGAGGA**·····**GGAAGTGGAGACAGACTGTC |
| **NPM1A P2** | Wild-type | TGTTGAGGAGCTCTGGAAGTGGAGACAGACTGTCAAATAAATG |
|  | Founder 1 | TGTTGAGGAGCTCTGGA**·······**ACAGACTGTCAAATAAATG |
|  | Founder 2 | TGTTGAGGAGCTCTGG**···**TT**···**ACAGACTGTCAAATAAATG |

The sequence of one single mutant F1 embryo from each founder is shown. Underlined text represents TALEN binding sites, dots represent deletions and boxed nucleotides represent insertions.
