# Supplementary material for: High Efficiency In Vivo Genome Engineering with a Simplified 15-RVD GoldyTALEN Design
Source: PLoS One. 2013 May 29;8(5):e65259. doi: 10.1371/journal.pone.0065259 (PMC3667041; doi:10.1371/journal.pone.0065259)
Supplement: Table S4 — Specificity of 15-RVD GoldyTALENs. (DOC) [file pone.0065259.s007.doc]

**Supplementary Table S4. Specificity of 15-RVD GoldyTALENs.**

| **TALEN Pair** | **Non-specific binding in zebrafish genome*** | | |
| --- | --- | --- | --- |
|  | 0 mis-match | 1-2 mis-match | 3-5 mis-match |
| FLT3 P1 | 0 | 0 | 0 |
| FLT3 P2 | 0 | 0 | 0 |
| FLT3 P3 | 0 | 0 | 0 |
| GFP(GM2) P1 | 0 | 0 | 0 |
| IDH1 P1 | 0 | 0 | 0 |
| IDH1 P1 RM | 0 | 0 | 0 |
| JAK2A P1 | 0 | 0 | 0 |
| JAK2A P1 LM | 0 | 0 | 0 |
| JAK2A P2 | 0 | 0 | 0 |
| JAK2A P3 | 0 | 0 | 0 |
| JAK2A P4 | 0 | 0 | 0 |
| JAK2A P5 | 0 | 0 | 0 |
| NPM1A P1 | 0 | 0 | 0 |
| NPM1A P2 | 0 | 1 | 0 |
| NPM1B P1 | 0 | 1 | 0 |
| NPM1B P1 LS | 0 | 0 | 0 |
| NPM1B P1 RS | 0 | 1 | 0 |
| NPM1B P2 | 0 | 1 | 0 |

*Predicted non-specific binding sites are defined by any off-target returns with product size between 30 and 100 bp from NCBI Primer-BLAST against the consensus zebrafish genome. The two TALEN binding sequences (reverse complementary for the right arm) were input as primer queries. Any returns with a product size <30bp or >80 bp means the two TALEN arms are not separated with any spacer sequence or are separated by >50bp spacer and therefore should not have any activity.
