# Supplementary material for: High Efficiency In Vivo Genome Engineering with a Simplified 15-RVD GoldyTALEN Design
Source: PLoS One. 2013 May 29;8(5):e65259. doi: 10.1371/journal.pone.0065259 (PMC3667041; doi:10.1371/journal.pone.0065259)
Supplement: Table S5 — Primer sequences used in this study. (DOC) [file pone.0065259.s008.doc]

**Supplementary Table S5. Primer sequences used in this study.**

| **Primers** | **Sequence** | | | **Annealing Temperature (ºC)** | |
| --- | --- | --- | --- | --- | --- |
|  | **Forward** | **Reverse** | |  |  |
| **RFLP assay for small INDEL** | | | | | |
| FLT3 P1 | TGAAAGTCTTCTTGCCTCTGTTC | | CAGCTGTAAATGAGTCTCACAGTT | | 60 |
| FLT3 P2 | TCACTGAAGGACAAGTGGTGG | | TCCCAGAGCATTACTGGCAC | | 61 |
| FLT3 P3 | CAAGCCTGATCTCACAAGGA | | GATCTCCCAGAGCAGAATGC | | 60 |
| GFP(GM2) | TCACTGGAGTTGTCCCAATT | | GTCTGCTAGTTGAACGCTTCC | | 55 |
| IDH1 P1 | GCTGGGGACCTATTCTACCC | | CCGGCAGTGATAAGCGTAAT | | 58 |
| JAK2A P1 | TGGGCGCTGCTTCAGATAAC | | CACGCTGTCTGACCTGATGA | | 58 |
| JAK2A P2 | TGGGCGCTGCTTCAGATAAC | | CACGCTGTCTGACCTGATGA | | 58 |
| JAK2A P3 | TGGGCGCTGCTTCAGATAAC | | GCGGAGCTACAGACAGTGAA | | 60 |
| JAK2A P4 | GCTGATGGTTATTGCTTTCTCTC | | ATCATCGTTTGCTGCTCACCA | | 61 |
| JAK2A P5 | GCTGATGGTTATTGCTTTCTCTC | | ATCATCGTTTGCTGCTCACCA | | 61 |
| NPM1A P1 | TGCTCACGGTTCACTTCTCC | | TCGAGGAATGGCACGGATTT | | 64 |
| NPM1A P2 | TGCTCACGGTTCACTTCTCC | | TCGAGGAATGGCACGGATTT | | 64 |
| NPM1B P1 | ACAAGGCATGCGAGTCTTCA | | ATCTGTAACCCCTGCCGAGG | | 60 |
| NPM1B P2 | ACAAGGCATGCGAGTCTTCA | | ATCTGTAACCCCTGCCGAGG | | 60 |
|  |  | |  | |  |
| **PCR screening for large deletion** | | | | | |
| FLT3 P1/P3 | TGAAAGTCTTCTTGCCTCTGTTC | | GATCTCCCAGAGCAGAATGC | | 60 |
| JAK2A | TGGGCGCTGCTTCAGATAAC | | ATCATCGTTTGCTGCTCACCA | | 60 |
|  |  | |  | |  |
| **qPCR to quantify large deletion** | | | | | |
| FLT3 P1/P3 | GCATGACAAGTAGTGCGTGT | | TCCCAGAGCATTACTGGCAC | | 60 |
| JAK2A P1/P4 | CGCTTCTGAGTGAGTGGAGA | | TATGTGCTCAGATCAGCTCCA | | 60 |
