# Supplementary material for: High Efficiency In Vivo Genome Engineering with a Simplified 15-RVD GoldyTALEN Design
Source: PLoS One. 2013 May 29;8(5):e65259. doi: 10.1371/journal.pone.0065259 (PMC3667041; doi:10.1371/journal.pone.0065259)
Supplement: Table S6 — Highest tolerable injection dosage of TALEN mRNA. (DOC) [file pone.0065259.s009.doc]

**Supplementary Table S6. Highest tolerable injection dosage of TALEN mRNA.**

| **TALEN Pair** | **Highest tolerable dosage* (pg)** |
| --- | --- |
| FLT3 P1 | 60 |
| FLT3 P2 | 60 |
| FLT3 P3 | 60 |
| FLT3 P1/P3 | 30 |
| GFP(GM2) P1 | 60 |
| IDH1 P1 | 60 |
| IDH1 P1 RM | 60 |
| JAK2A P1 | 80 |
| JAK2A P1 LM | 80 |
| JAK2A P2 | 40 |
| JAK2A P3 | 20 |
| JAK2A P4 | 60 |
| JAK2A P5 | 80 |
| JAK2A P1/P4 | 30 |
| NPM1A P1 | 60 |
| NPM1A P2 | 60 |
| NPM1B P1 | 60 |
| NPM1B P1 LS | 60 |
| NPM1B P1 RS | 60 |
| NPM1B P2 | 60 |

*Amount of mRNA injected for each arm of 1 or 2 TALEN pair(s).
